# Neoteric Biofilms Applied to Enhance the Safety Characteristics of Ras Cheese during Ripening

**DOI:** 10.3390/foods12193548

**Published:** 2023-09-24

**Authors:** Rasha A. Ibrahim, Baraka A. Abd El-Salam, Tawfiq Alsulami, Hatem S. Ali, Karolina Hoppe, Ahmed Noah Badr

**Affiliations:** 1 Dairy Research Department, Food Technology Research Institute, Agricultural Research Centre, Giza 12619, Egypt; ra.ibrahim@arc.sci.eg (R.A.I.);; 2 Food Science & Nutrition Department, College of Food and Agricultural Sciences, King Saud University, Riyadh 11451, Saudi Arabia; 3 Food Technology Department, National Research Centre, Cairo 12622, Egypt; hatem.owyean1@gmail.com; 4 Chemistry Department, Poznan University of Life Science, ul. Wojska Polskiego 75, 60-625 Poznan, Poland; 5 Food Toxicology and Contaminants Department, National Research Centre, Cairo 12622, Egypt

**Keywords:** aflatoxins, pathogens, toxigenic fungi, cell-free extracts, cheese rheology, bacterial pellet, sensory evaluations

## Abstract

The milk’s natural flora, or the starter, can preserve cheesemaking and allow for microbial competition. This investigation aimed to improve cheese safety and assess its characteristics using probiotic cell pellets (LCP) or cell-free extracts (CFS). Cheese samples were collected from different areas to investigate the current contamination situation. Six CFSs of probiotics were assessed as antifungal against toxigenic fungi using liquid and solid media and their aflatoxin reduction impact. The most effective CFS was chosen for cheese coating in nanoemulsion. Coated cheese with CFS, LCP, and LCP-CFS was assessed against control for changes in chemical composition, ripening indications, rheological properties, and microbiology. Results showed significant contamination levels in the collected samples, and toxic fungi were present. *Lactobacillus rhamnosus* CFS has aflatoxins reducibility in liquid media. During cheese ripening, uncoated cheese showed higher fat, protein, salt content, soluble nitrogen, total volatile fatty acids, tyrosine, and tryptophan contents than coated samples, except for LCP-coating treatment. Cheese rheology indicated that coating treatments had the lowest hardness, cohesiveness, gumminess, chewiness, and springiness compared to uncoated cheese. Uncoated cheese had the highest yeast and mold counts compared to the treated ones. The LCP-CFS-coated cheese showed no *Aspergillus* cells for up to 40 days. Uncoated Ras cheese recorded slightly lower flavor, body, texture, and appearance scores than coated cheeses. In conclusion, coating cheese with *L. rhamnosus* nanoemulsion has antifungal and antiaflatoxigenic properties, even for LCP, CFS, and CFS-LCP, which could extend cheese shelf life.

## 1. Introduction

Ras cheese is the famous hard type of cheese produced in Egypt, made from raw cow milk or a mixture of cow and buffalo milk. It exhibits similarities to the Greek cheese variety known as Kefalotyri. The terminology in both nations signifies “cranium”. It is essential to mention that Ras cheese is only documented in scientific literature, while it is known in terms of Romi or Torky cheese in markets. The basic Ras cheese is believed to have originated in the Balkans and then spread to Egypt during the early phase of the Egyptian Industrial Renaissance [1]. Ras cheese is usually stored in moist and uncontrolled hygienic conditions, supporting mold growth and yeasts [2]. Mold species are responsible for many severe diseases by producing toxic metabolites called mycotoxins [3]. Cheese contamination by molds may occur from the raw material (milk) or during manufacturing, storage, and distribution [4]. Certain fungi naturally produce mycotoxins, causing disease and death in humans and other animals [5]. Various *Aspergillus* species produce aflatoxins (AF), which are highly toxic carcinogenic compounds causing food safety reduction [6]. Ingestion of high aflatoxin levels can cause acute toxicity, leading to a severe illness or even death. Long-term exposure causes cancer, immune suppression, and liver damage [7]. There is great demand for aflatoxin control to ensure the safety of products and consumers’ health [8].

One of the traditional approaches for solving the problems associated with fungal decay in the cheese industry is the application of coatings based on paraffin, polyvinyl acetate, plasticizers, antibiotics (natamycin), and other chemical substances to protect the cheese surface [9]. However, the wide use of chemical preservatives and fungicides risks the consumer’s health, although most can be used in foods. Therefore, applying coatings enriched with natural and safe components could avoid or delay fungal contamination, improving the safety characteristics of hard-cheese types during ripening. Utilizing composites in cheese coatings, such as carboxymethyl cellulose (CMC) and chitosan, which are loaded with natural extracts, has a protective effect [10,11]. Also, lactic acid bacteria were reported to have antifungal and antimycotoxigenic impacts by their application in cheese manufacturing [12,13,14]. At the same time, their loading as encapsulating bacterial cells or their metabolites could reduce the toxicity of aflatoxin M1 in dairy products [15].

There has been a growing interest in using lactic acid bacteria (LAB) and other natural alternatives without chemical additives to counteract food toxins. Their non-pathogenic and non-toxic nature allow many LAB applications to improve safety and quality in food processing [16]. Lactic acid bacteria are known to inhibit mold growth and, to some extent, bind aflatoxins in different matrices [17]. Lactic acid bacteria can eliminate or remove mycotoxins in food through physical attachment or bio-transforming mechanisms [18]. Cell-free bacteria (CFS) extracts are solutions derived from bacterial cultures and contain a mixture of bioactive compounds, such as enzymes, antimicrobial peptides, and metabolites [19]. These extracts have been studied for their potential use in food preservation, as they can provide a natural alternative to synthetic preservatives. CFSs have been shown to have antimicrobial activity against various foodborne pathogens, including *Listeria monocytogenes* Scott A, *Salmonella typhi* ATCC 14028, and *E. coli* ATCC 25922. They can also inhibit the growth of spoilage microorganisms, which can help extend food products’ shelf life [20].

Not all probiotic strains effectively prevent mycotoxin contamination, and the effectiveness can depend on the type of mycotoxin and the cheese-making process. An earlier study reported that the capability of some probiotic strains was significantly better than that of commercial starter strains [21]. Although typical starters are required for the product’s aroma and acceptability, other strains for safety requirements can be applied at the outer surface. The present study was intended to improve the safety characteristics of Ras cheese during its ripening process. Incorporating bacterial cells singly or with their metabolites to coat cheese can protect against microbial contamination during its storage for ripening. This function is achieved using edible coatings enriched with lactic acid bacteria to reduce human health risks.

## 2. Materials and Methods

### 2.1. Materials, Chemicals, and Microorganisms

Samples of Ras cheese were collected from six geographical regions, mainly the producing areas of Egypt, to sample the actual contamination values. Isolated microorganisms were identified, and fungal strains were reused to examine the suggested treatment for safety production. All chemicals, media, and indicators in this experiment were from Oxoid, while solvents, reagents, and standards are from Merck’s analytical grade.

Six lactic acid bacteria (LAB) strains were gifted from the Dairy Research Department, Food Technology Research Institute, Agricultural Research Centre, Giza, Egypt. These strains are *Lactobacillus acidophilus* ATCC 4356, *Bifidobacterium bifidum* NCTC 13001, *Lactobacillus plantarum* ATCC 14917, *Lactobacillus rhamnosus* GG^®^, *Lactobacillus helveticus* ATCC 10386, and *Lactobacillus casei* ATCC 7469. Fungal strains of *Aspergillus flavus* ATCC 9643, *Aspergillus niger* ATCC 6275, *A.paraciticus* ATCC 15517, *A. nominos* ATCC 208928, *Penicillium chrysoginum* ITEM 4680, and *P. notatum* ATCC 10106 were isolated from collected Egyptian cheese samples. The high-aflatoxin-producing strain of *Aspergillus flavus* ITME 698 was obtained from ISPA, Institute of the Sciences of Food and Production, Bari, Italy.

### 2.2. Microbiological Analysis of Collected Cheese Samples

The total microbial count was determined according to the methodology described by Şengül [22] with modifications. Briefly, a 20 g cheese in 100 mL (2.0%) sodium citrate solution was homogenized (5 min) using a Lab-Blender with a stainless steel cup (Waring Lab Co., Maple St, Carrollton, TX, USA; Model number 7010HS). Serial dilutions were prepared using 0.1% sterile peptone water. The following analyses were made on cheese samples: total colony counts (PCA; 37 °C/48 h); coliforms on violet red bile agar (VRBA; at 32 °C/24 h) [23]; Staphylococcus aureus on Baird–Parker egg yolk–tellurite medium (BPM; at 37 °C/48 h) [23]; total LAB on the Elliker agar at 37 °C/24 h; molds and yeasts on yeast extract sucrose agar (YESA; at 25 °C/5 days) [24].

Biochemical identification insurance for gifted strains was made for the bacterial strains using the API-strip test (Analytical Profile Index, bioMérieux-Boston, MA, Cambridge, USA) to emphasize the purity, where the techniques used have been described in detail previously by Funke et al. [25]. The commercial API kits (bioMérieux, Marcy l’Etoile, France) were used according to the manufacturer’s instructions, and reading was performed after incubation.

### 2.3. Fungal Isolation and Identification

Cheese samples were cut into 5 mm in diameter using a sterilized cutter. The surfaces of the slices were sterilized using a hypochlorite solution (2% for 2 min). The cheese slices were washed with autoclaved water and plated aseptically on Sabouraud dextrose agar (SDA) media. The cultured plates containing tested samples were then incubated (at 25 °C/5 days).

A pure culture of each fungal strain was obtained by single spore technique and maintained by sub-culturing each colony that emerged onto the SDA plates, where it was identified. The fungal isolates were identified using cultural and morphological features such as colony growth pattern, conidial morphology, and pigmentation [26]. The technique of Oyeleke and Manga [27] was also adopted for identifying the isolated fungi using cotton blue in lacto phenol stain. The identification was achieved by placing a drop of the stain on the clean slide with a mounting needle. A small portion of the representative fungi cultures’ aerial mycelia was spread in the lacto phenol stained. The mycelia of the fungi were well distributed on a slide using a needle. A cover slip was gently placed with little pressure to eliminate air bubbles. The microscopic slide was then mounted and viewed under the light microscope with objective lenses. The morphological characteristics and appearance of the fungal organisms seen were identified following Adebayo-Tayo et al. [28].

### 2.4. Preparation of Cell-Free and Cell-Solution from the LAB Strains

According to the previous methodology, the cell-free supernatant (CFS) was prepared [14,29]. The solution derived from the LAB strains pellet was collected, purified, and sterilized by an individual sterile membrane (0.22 μm) and stored until the application in diffusion assays. The LAB cell–pellet (LCP) collected sediment was prepared according to El-Nezami et al. [30,31]. Hydrochloric acid (1 M) was used for acidifying the pellet–cell precipitate. The bacterial concentrations for this step ranged between 2.6–2.9 × 10^10^ CFU/mL media.

### 2.5. Determination of the LAB Extracts’ Antifungal Effect

The antifungal activity of the CFSs was screened by agar diffusion assay (disc and well assays) according to CLSI methodology [32]. The CFS of each LAB strain was assessed individually on the agar media. Fungal strains were reactivated on Czapek–Dox agar supplemented with tetracycline to suppress bacterial growth. The effect is expressed as an inhibition zone surrounding the disk or the well (mm). The more inhibition zone area, the more significant the strain affected.

### 2.6. Determination of the Antimycotic and Antiaflatoxigenic Inhibitory Effect

Liquid media were utilized following the approach of Shehata et al. [29] to evaluate the inhibiting activity against toxigenic fungal growth. The treatments’ inhibitory and reduction effects were assessed for the mycelial growth weight and aflatoxin production of *Aspergillus flavus* ITME 698 using liquid yeast extract sucrose media. One-liter flasks containing 250 mL of media were utilized for the experiment. The fungi-treated flasks were divided into six groups, the first being the control group. Each fungus’ growth inhibition was estimated as the decrease happened in the mycelia dry weight of each treatment against the control of the same fungus.

### 2.7. Determination of Aflatoxin Reduction Effect

The filtrated media from mycelia weight evaluation of the *Aspergillus* strain were chosen to examine the presence of aflatoxins (AFB_1_, AFB_2_, AFG_1_, and AFG_2_). The quantitative analyses utilized a pre-calibrated fluorometer (VICAM Series 4EX Fluorometer, Watertown, MA, USA; LOD 1.0 ng/Kg). According to the methodology described by Farouk et al. [33], 1 mL of the solution was mixed with 10 mL of distilled water before being deposited on an Afla-test immunological affinity column and rinsed twice with 10 mL of distilled water (flow rate: 6 mL/min). A 2 mL of methanol was applied to the Afla-test column (flow rate: 0.5 mL/min) to elute AF contents. The elution was then determined for the AF content using a VICAM fluorometer.

### 2.8. Preparation of Nanoemulsion for Ras Cheese Coating Film

The data from previous evaluations indicated the higher antifungal activity as the *L. rhamnosus* strain. This strain was chosen to enhance the safety production of Ras cheese. Two forms of applications were applied (CFS and LCP). Besides their mix (CFS-LCP), these materials were prepared in the form of nanoemulsion.

Chitosan powder (Sigma-Aldrich^®^ Solutions, Merck Life Science, Merck KGaA, Darmstadt, Germany) was utilized to prepare a solution of 2% concentration. The extract-loaded chitosan nanoemulsion was made using a modified approach from prior research [34]. In brief, chitosan solution (2%; *w*/*v*) was created by dissolving chitosan powder in an aqueous lactic acid solution (1%; *v*/*v*) overnight at room temperature. Tween 80 was added to the solution and agitated (4 h/60 °C) for a homogenous mixture. After complete dissolution, glycerol (1%, *v*/*v*) was added and stirred continuously (4 h). Sodium tri-poly phosphate solution (0.1%; *w*/*v*) was progressively added to the emulsion while mixing; agitation was continued (1 h).

The extract was added gradually to the swirling mixture and agitated (10 min). The pH value of 5.4 for the final solution was adjusted. Three loading-coat materials were prepared using chitosan solution, using CFS, LCP, and their mix (1:1). The Ultra-Turrax (T 25 digital Ultra-Turrax^®^, IKA^®^-Werke GmbH & Co., Janke & Kunkel-Str., Staufen 79219, Germany) was then used to homogenize the solution. After that, the solution of each extract concentration was exposed to ultrasonication treatment (ultrasonication at 160 W powers, 20 kHz frequency, 50% pulse, Sonic Ruptor 400, OMNI International).

### 2.9. Determination of Coating Film Characterization

Nanoemulsion was evaluated for the characterization of particle size (PSS), zeta potential value (ZPV), and poly dispersing index (PDI) using the same methodology described by Malik et al. [35]. The Malvern apparatus (Nano-S90, Zetasizer, Malvern Panalytical Ltd., Enigma Business Park, Grovewood Road, Great Malvern, UK) was utilized to estimate the PSS, ZPV, and PDI values. The emulsion was transferred to a 20 mL cylinder, capped, and stored for 24, 48, 72, 96, and 120 h at 25 °C [36]. The serum separation from the emulsion will be the emulsion stability index. The height of separated serum from the emulsion monitored % separation is calculated using the following equation:

%SR=(H1/H0)×100

where *H*1 is the upper phase height;

*H*0 represents the initial emulsion height;
*SR* is the stability ratio for the formed emulsion.

The surface characteristics of films were evaluated using a transmission electron microscope according to the methodology and apparatus described before [37].

### 2.10. Ras cheese Making

A mixture of raw cow and buffalo milk (3:2, 4.8% fat, and 0.18% acidity as lactic acid) was heated at 63 °C/30 min and then cooled to 40 °C. Thermophilic yogurt culture YC-X11 (3%, *w*/*w*) was added. After one hour of milk ripening at 40 °C, the appropriate amount of Chymosin was added to clot the milk in 40 min. The coagulum was cut and cooked to 45 °C/45 min and held at this temperature for 15 more minutes. The curd was salted (3%, *w*/*w*, NaCl), hooped, and pressed as described by Hofi et al. [38]. The resultant cheese wheels were placed in brine (20% NaCl solution) for the proposed period at 10 °C. Cheese wheels were divided into five groups: control (T1, without coating film); T2, coated with coating film without any additions; and the other three treatments (T3, T4, T5) were covered with coating films supplemented with cell-free supernatant (CFS), LAB cell-pellets (LCP) of *L. rhmnosus* or their mix, respectively. Two wheels were made for each treatment. Cheese treatments were stored at 13 ± 2 °C and 80 ± 5% relative humidity for 120 days and sampled at fresh and monthly intervals for up to four months. Three replicates were performed for every treatment.

### 2.11. Experimental Design for the Bacterial Application in Cheese Safety Production

The experimental simulations for Ras cheese coating using chitosan-loaded film of the LCP, the CFS, and CFS-LCP were performed against the control. Samples were stored for maturation, swapped every 15 days, and inspected for each treatment to determine the presence of *Aspergillus flavus* contamination. The results were expressed (log CFU/g ± SD). The experimental simulations for Ras cheese coating using chitosan-loaded film of the LCP, the CFS, and CFS-LCP were performed against the control.

### 2.12. Analytical Methods of Ras Cheese Treatments

#### 2.12.1. Chemical Composition

Methods of AOAC [32] determined moisture, titratable acidity, fat, Ash, and protein contents. The pH was determined using a digital pH-meter HANNA 213 (Viale delle Industrie, Villafranca, Padovana, Italy). The salt content was determined using the method of Bradley et al. [39].

#### 2.12.2. Ripening Indices

Soluble nitrogen (SN) was determined according to Ling [40]. Total volatile fatty acids (TVFA) were determined according to the method described by Kosikowski [41]. The value was expressed as ml of 0.1 N NaOH/100 g cheese. Cheese samples’ soluble tyrosine and tryptophan contents were determined according to Vakaleris and Price [42].

#### 2.12.3. Texture Profile Analysis

Texture profile analysis (TPA) was performed using at least three samples for each treatment with Universal Testing Machine (Cometech, B type, Taiwan) provided with the software. A back extrusion cell with a 35 mm diameter compression disc was used. Two cycles were applied at a constant crosshead velocity of 1 mm sec G1 to 35% of sample depth, then returned the resulting force–time curve.

#### 2.12.4. Microbiological Changes during the Ripening Period

Cheese samples were examined for the total bacterial count as a method recorded by Laird et al. [43]; yeast and mold counts were recorded according to the IDF 94A method [44], and the total proteolytic or lipolytic bacterial count was carried out as described by Luk [45]. *Aspergillus flavus* count of cheese samples was carried out every 20 days according to a method of Duan et al. [46], using Rose Bengal chloramphenicol agar and incubated for seven days at 25 °C. The results were expressed as log CFU/g ± SD.

#### 2.12.5. Sensory Properties of Ras Cheese Treatments

According to Pappas et al. [47], the sensory properties of the cheese samples were judged by 15 panelists for appearance, flavor, body, and texture using a sensory evaluation sheet.

### 2.13. Statistical Analysis

All the experiments were performed in five replicates, and the data were presented as means with standard deviation (mean ± SD). Values were compared using the ANOVA test, and Duncan’s post hoc test was performed to determine the significance of values. The statistical analysis was performed using the SPSS 16.0 software program.

## 3. Results

### 3.1. Evaluation of Current Contamination

The data in supplementary Table S1 reflect the microbial contamination in collected cheese samples present in this investigation. Twelve microorganisms were identified and isolated (six bacteria and six fungi). The bacterial content of examined samples includes four pathogenic bacterial strains, including *E. coli*, *Staphylococcus aureus*, *Salmonella typhi*, and *Bacillus subtilis*. Isolated fungal strains from cheese samples include four *Aspergilli* and two *Penicillium* species. At the level of the cheese samples collected for the study, the results showed a high content of contamination with pathogenic bacterial strains (four strains) in the samples taken from the eastern and western Delta regions, with also high levels of contamination with both *Aspergillus* and *Penicillium* fungi (six strains). The cheese samples collected from the Upper and Lower Egypt regions showed a greater dominance of fungal contamination compared to a lower percentage of bacterial contamination (Table S1). This may be explained by competition between microbes and according to the optimal environmental conditions for each species in terms of temperature and relative humidity. It was noted that bacterial contamination decreased compared to fungal contamination whenever the samples were collected from the southern regions, except for *Staphylococcus aureus* bacteria, which recorded an increase in those samples. This increase in bacterial numbers of *Staphylococcus* can be explained by accidental contamination during ripening and storage.

### 3.2. Antifungal Effect of LAB Extracts

**Figure 1:**
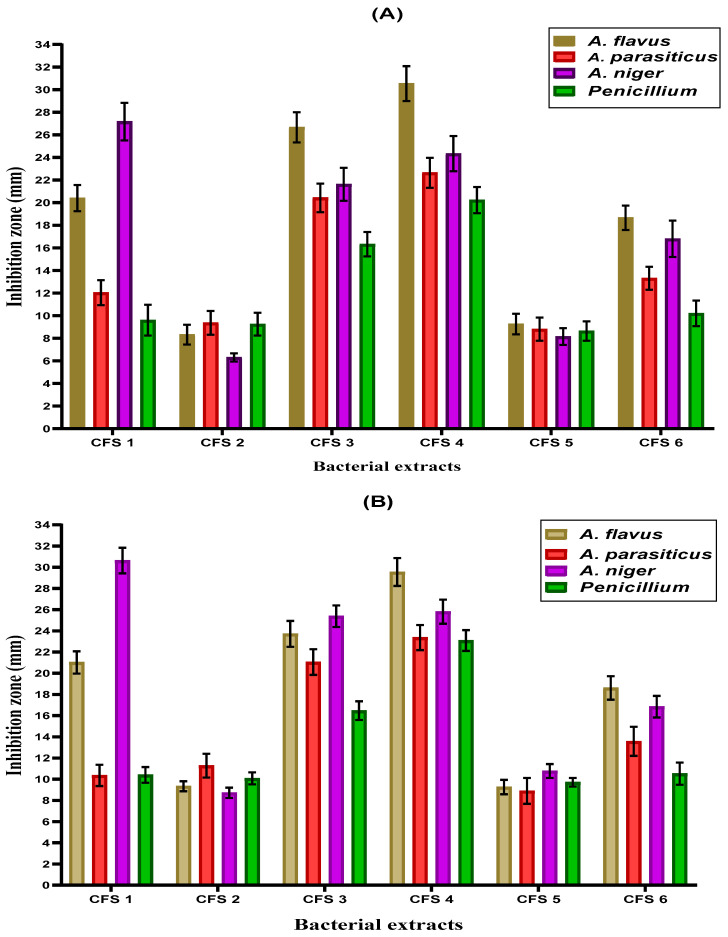
Antifungal effect of lactic acid bacteria cell-free extracts determined by two diffusion assays. Results expressed as mean ± SD; (**A**) represents the disc diffusion assay; (**B**) represents a well-diffusion assay CFS 1: cell-free extract of *L. acidophilus*; CFS 2: cell-free extract of *B. bifidum;* CFS 3: cell-free extract of *L. plantarum;* CFS 4: cell-free extract of *L. rhamnosus;* CFS 5: cell-free extract of *L. helveticus;* CFS 6: cell-free extract of *L. casei*.

The antifungal activity of lactic acid bacteria extracts (the cell-free supernatant (CFS) against fungal isolates using two diffusion assays (disc and wells) is illustrated in Figure 1A,B. It could be seen that the cell-free extract of *L. rhamnosus* (CFS 4) had the highest inhibitory effect against all tested fungal strains compared to other cell-free extracts, followed by the cell-free extract of *L. plantarum* (CFS 3) in both two diffusion assays (disc and wells). *Aspergillus flavus* and *A. niger* were more susceptible to most cell-free extracts than other tested fungal strains. The results of both diffusion assays (disc and wells) are approximately similar.

The inhibition of toxigenic fungal growth was significant regarding two of the CFSs that belonged to *B. bifidum* and *L. helveticus*. For the CFS obtained from *L. acidophilus*, the antifungal impact varied between the applied strain, where the inhibition was high against *A. paraciticus* and *Penicillium* fungi.

### 3.3. Mycelial Dry Weight

**Figure 2:**
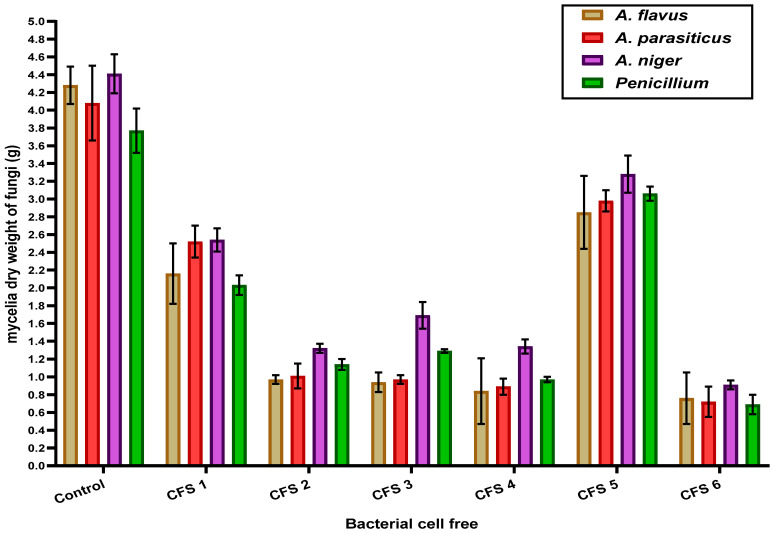
Influence of the cell-free supernatant of lactic acid bacteria on the dry weight of fungal mycelia growth (g) in liquid media. Results expressed in means± SD (n = 5; *p* = 0.05); CFS 1: cell-free extract of *L. acidophilus*; CFS 2: cell-free extract of *B. bifidum*; CFS 3: cell-free extract of *L. plantarum*; CFS 4: cell-free extract of *L. rhamnosus*; CFS 5: cell-free extract of *L. helveticus*; CFS 6: cell-free extract of *L. casei*.

To ensure the antifungal activity of the cell-free supernatant, it was also evaluated using liquid growth media of fungi. In this assay, the inhibition was determined depending on the reduction of the mycelia weight of each treated fungi against the control. The data in Figure 2 represent the mycelia dry weight (g) of each fungus in control and by the cell-free application in liquid media of fungal growth (100µL/mL media) as affected by lactic acid bacteria extracts (the cell-free supernatant (CFS)). The lowest values of tested fungi mycelia dry weight were recorded with a cell-free extract of *L. casei* (CFS 6) followed by a cell-free extract of *L. rhamnosus* (CFS 4). Also, it could be observed that the cell-free extract of *L. helveticus* (CFS 5) had the lowest inhibitory effect on the growth of all investigated toxigenic fungi. *A. niger* is the most resistant to all the tested cell-free supernatants.

### 3.4. Aflatoxins Production

**Figure 3:**
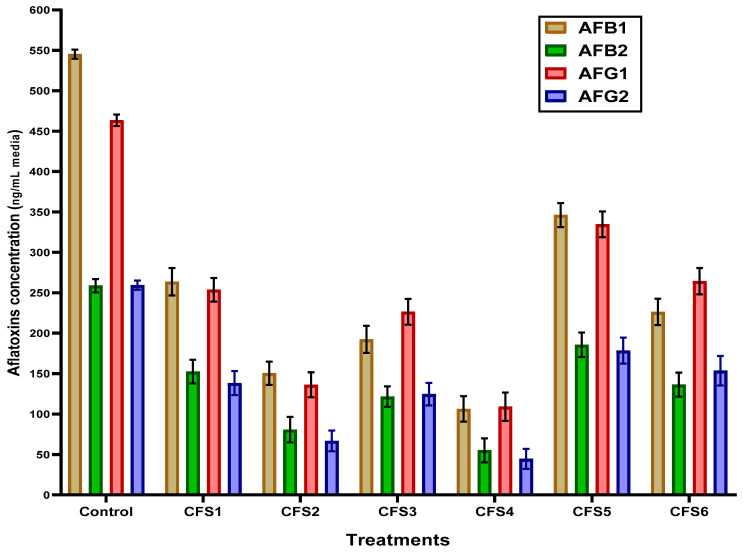
Determination of aflatoxin production in liquid media of *Aspergillus flavus* ITME 698 in the presence of bacterial cell-free extract against the control. Results are expressed in means ± SD (n = 5; *p* = 0.05); AFB_1_: aflatoxin B_1_; AFB_2_: aflatoxin B_2_; AFG_1_: aflatoxin G_1_; AFG_2_: aflatoxin G_2_; CFS 1: cell-free extract of *L. acidophilus*; CFS 2: cell-free extract of *B. bifidum;* CFS 3: cell-free extract of *L. plantarum;* CFS 4: cell-free extract of *L. rhamnosus;* CFS 5: cell-free extract of *L. helveticus;* CFS 6: cell-free extract of *L. casei*.

While fungal growth reduction is significant for product safety, the contamination by their metabolites is also significant for safety. For this reason, cell-free extract’s impact on reducing the fungal ability of toxin production was assessed. The aflatoxin production results in liquid media of *Aspergillus flavus* in the presence of bacterial cell-free extracts compared to control are presented in Figure 3. The production of different types of aflatoxin (aflatoxin B_1_, aflatoxin B_2_, aflatoxin G_1_, aflatoxin G_2_) from *A. flavus* decreased in the presence of all tested bacterial cell-free extracts. The lowest values of different aflatoxins were recorded with a cell-free extract of *L. rhamnosus* (CFS 4). Aflatoxin production in control was very high compared to treatments with bacterial cell-free extracts.

### 3.5. Characteristics of the Emulsions Used in the Coating Process

The mean sizes of nanoparticles formed from the CFS, LCP, and their mix are shown in Table 1. Other characteristics indicate emulsion stability, including nanoemulsions and the poly dispersing index (PDI), z-potential values, and stability index. The droplet size of the chitosan emulsion was recorded as mean as 102.84 ± 4.18 nm; this value was acceptable compared to the loaded emulsion types of the CFS, the LCP, and the CFS-LCP composites. A significant advantage of nanotechnology is its ability to design and enhance the distinctive physicochemical properties of small composite structures. Changing size, shape, or surface chemistry can regulate a nanoparticle’s functionality.

**Figure 4:**
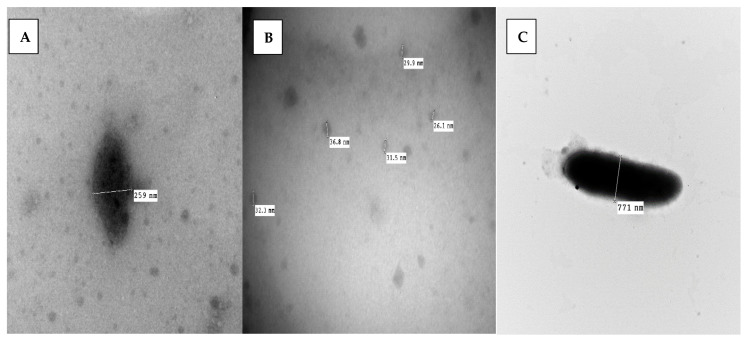
Transmitted electron microscope capture of bacterial forms applied to form coating nanoemulsion. (**A**): LCP-nanoemulsion of the; (**B**): CFS-nanoemulsion; (**C**): CFS-LCP mix nanoemulsion. CFS: cell-free supernatant; LCP: bacterial cell pellets; Mix CFS-LCP: a solution containing cell pellets and supernatant as a mix.

The parameter of Zeta potential plays a significant role in the overall stability of the emulsion. As shown in Table 1, the recorded PDI values ranged between 0.37 ± 0.11 to 0.49 ± 0.04 mV, reflecting the strength of formed emulsions and the low aggregated properties of the particles contained in the emulsions. High ratios reflect the stability recorded for the formed emulsion for the examined types of emulsion. Nanoemulsion stability could be achieved even with zeta potential less than (+20 mV) or (−20 mV) by changing surface tension, which occurred in the present emulsion due to polysorbate. It was also characterized using the transmitted electron microscope to ensure the emulsion formation, where captures are recorded in Figure 4.

### 3.6. Evaluation of Formed Films in Ras Cheese Coating

#### 3.6.1. Chemical Composition of Ras Cheese Treatments

The chemical composition of the coated Ras cheese treatments is given in Table 2. The moisture of the control or different covered Ras cheese treatments decreased by progressing ripening periods. Still, this increase was lower for the coated cheese treatments than for the control (non-coated). The fat content of all Ras cheese treatments increased with increasing ripening periods. The lowest fat content of different coated Ras cheese treatments was observed in sample T4 (bacteria + CFS) (33%, *w*/*w*).

Also, it could be clear from the data that the protein content increased by progressing the ripening periods in control (non-coated) or treatments with different coating materials. The lowest protein content was recorded at the end of ripening periods with T2 (cheese coated by bacteria), probably due to the proteolytic activity of bacteria in the cheese coat. The increase in protein and fat contents in all treatments during storage was mainly linked to moisture loss. Table 2 shows that fat/dry matter increased by progressing the storage period. There were no significant differences in the salt content of different Ras cheese treatments and control at zero time of storage period. Salt content increased over the storage period for other coated cheese and control, probably due to the water loss caused by evaporation. The highest salt level was observed with control non-coated). Increasing storage period increases cheese acidity; this increase in lactic acid is also directly related to moisture loss. The highest value of acidity was recorded with cheese coated with bacteria. Oppositely, the changes in pH values among all studied Ras cheese treatments during ripening periods showed an opposite trend to those of titratable acidity. Milk constituents naturally cause the total acidity of cheese in addition to acidity developing during cheese ripening. The degradation intermediate compounds of protein and amino acids and fatty acids resulting from fat hydrolysis would appreciably contribute to an increase in cheese acidity.

#### 3.6.2. Ripeni006Eg Indices of Ras Cheese

The ripening indices of Ras cheese during the ripening period are indicated in Table 3. The soluble nitrogen (SN) of uncoated (control) or different coated Ras cheese treatments gradually increased overall ripening periods. The increase in soluble nitrogen of uncoated (control) was more than those of other covered Ras cheese treatments except for the treatment coated with *L. rhamnosus* pellets (T2), where the soluble nitrogen value of T2 was very close to the control. The highest SN value was noticed with control (0.74%), followed by T2 (0.72%). The SN value of coated cheese (T1) is lower than that of the control. The coated cheese proteolysis is slower than uncoated cheese. The production of soluble nitrogen compounds through cheese ripening indicates the proteolysis rates. It is an indicator of casein hydrolysis by the rennet action, starter culture enzymes, and milk proteases present at the beginning of the ripening period.

#### 3.6.3. Rheology Properties of Ras Cheese

Table 4 shows the rheology results of Ras cheese. The cheese texture is the combination of different parameters, including cohesiveness, Adhesiveness, gumminess, chewiness, springiness, and hardness, that play a role in the quality and consumer acceptance. Treatment of Ras cheese with formed films markedly affected the rheological properties. It could be seen from the data that the hardness value increased with the progress of the ripening period and reached the highest value at the end of the ripening period. The increase in the hardness values of the film treatments was less compared to the control. The lowest values of hardness were recorded for coated treatments that contained bacteria. The relationship between the cheese’s hardness and its moisture content is inverse.

A negative correlation was observed between adhesiveness and hardness or ripening period. The cohesiveness, gumminess, chewiness, and springiness followed the same trend of hardness and had the same differences in the uncoated and different coated Ras cheese. Higher hardness indicates that cheese has a tight inner structure, so its gumminess and cohesiveness are more significant. Hardness showed a high positive correlation with moisture, springiness, and chewiness. They stated that this is due to early casein matrix hydrolysis by residual enzymes in the rennet and also to the proteolytic system of added culture.

#### 3.6.4. Microbiological Changes in Ras Cheese

Microbiological changes in uncoated and different coated Ras cheese treatments are shown in Table 5. It could be seen from the data that the total bacterial count (TBC) of all investigated treatments increased up to 60 days after the ripening period and then decreased. It was observed that the TBC count of the uncoated cheese was lower compared to the coated cheese, and the most significant count was observed in cheese coated with bacteria (*L. rhamnosus* pellets). The coating of cheese increased the viability of lactic acid bacteria (starter and coating bacteria) compared to uncoated cheese. Concerning the yeasts and molds count, No yeasts and molds were detected at the beginning of the ripening period; it appeared from day 30 for uncoated cheese and increased until reaching the highest count at the end of the ripening period.

The times of yeast detection have differing values for the coated cheese treatments, where yeasts and molds appeared on 30, 60, and 90 days for T1 and T2, T3, and T4, respectively. The uncoated cheese had the highest yeast and mold counts compared to those of coated treatments; this is due to the low percentage of oxygen in the coated cheese, which are unsuitable conditions for the growth of molds. At the end of the ripening period, the T4 (Ras cheese coated with pellets and cell-free extract of *L. rhamnosus*) showed the lowest count of yeasts and molds. *Lactobacilli* bacteria create facultatively, which leads to the inhibition of molds. The lipolytic and proteolytic bacteria findings of uncoated and coated Ras cheese (Table 5) increased until the first and third months, respectively, then decreased. The highest count of proteolytic bacteria was recorded with T4 (Ras cheese coated with pellets and cell-free extract of *L. rhamnosus*).

**Figure 5:**
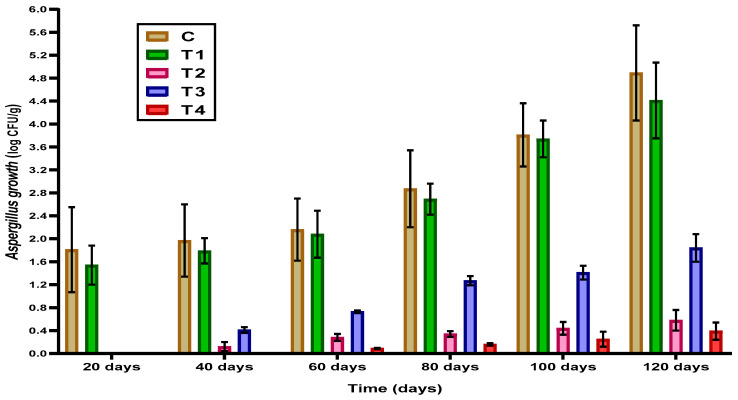
*Aspergillus flavus* count (log CFU/g) of uncoated and coated Ras cheese during the ripening period. The result was expressed in log CFU/g (mean ± SD; n = 5; *p* = 0.05); nd: represent undetected microbes in the examined samples, CFS: cell-free extract of *L. rhamnosus*; LCP: *L. rhamnosus* cell-pellets. C: control cheese, T1: cheese coated with plain film (a raw film without loading); T2: cheese coated with LCP; T3: cheese coated with CFS; T4: cheese coated with CFS-LCP.

The contamination of different coated cheese treatments was evaluated every 20 days, where the presence of *Aspergillus flavus* fungi was recorded (Figure 5). No *A. flavus* was detected in uncoated or different coated cheese treatments at the beginning of the ripening period. *A. flavus* was detected and highly increased in uncoated cheese by increasing the storage period, reaching the highest count at the end of the ripening period. Also, the data showed that *A. flavus* was detected on the 20th day for T 2 (Ras cheese with pellets of *L. rhamnosus*) and T3 (Ras cheese coated with cell-free extract of *L. rhamnosus*) and on the 40th day for T4 (Ras cheese coated with pellets and cell-free extract of *L. rhamnosus*), then increased till the end of ripening period. Cheese sample contamination can be viewed in Figure S1 of the Supplementary Materials.

The lowest counts of *A. flavus* were noticed for T4 (Ras cheese coated with pellets and cell-free extract of *L. rhamnosus*) during the ripening period. Probiotic bacteria can prevent mycotoxin contamination through various mechanisms. For example, some strains of probiotic bacteria can produce enzymes that degrade mycotoxins or bind to mycotoxins and prevent their absorption in the gut. Other strains of probiotic bacteria can compete with mycotoxin-producing molds for nutrients, space, and oxygen, thereby reducing the growth of these molds. In agreement with the present results, previous investigations have reported the probiotic bacteria utilization in cheese mycotoxin prevention. For example, *Lactobacillus* has been shown to inhibit the growth of *Aspergillus* in cheese. Other studies have shown that adding various probiotic strains to cheese can reduce aflatoxin and several mycotoxin levels.

#### 3.6.5. Sensory Properties of Ras Cheese during Ripening

**Figure 6:**
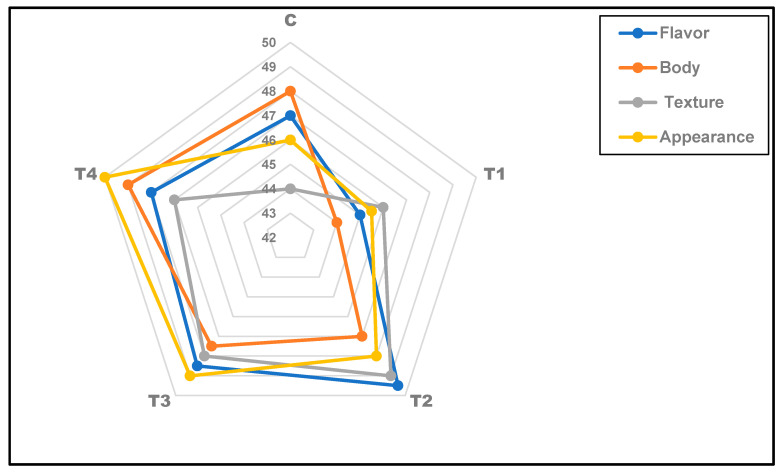
Overall sensory evaluation of coated Ras cheese samples compared to the control. C: control cheese; T1: cheese coated with plain film (a raw film without loading); T2: cheese coated with LCP; T3: cheese coated with CFS; T4: cheese coated with CFS-LCP.

The sensory scores of uncoated and different coated Ras cheeses at the ripening period are summarized in Figure 6: there were no conspicuous differences in flavor scores among all studied Ras cheese treatments. At the same time, the complete evaluations were recorded during the storage period and every 30 days up to 4 months. While uncoated Ras cheese recorded slightly lower flavor scores until the 90th day than those of coated cheeses, extensive growth of yeasts and molds appeared visually, which is unsuitable for sensory evaluation (Table 5 and Figure 5). The sensory evaluation results indicated that the different coated cheese treatments appeared better than those of uncoated Ras cheese until the end of the ripening period. T4 (Ras cheese coated with pellets and cell-free extract of *L. rhamnosus*) showed the best-studied sensory attributes. Coated cheese with chitosan recorded higher total organoleptic properties than uncoated cheese.

## 4. Discussion

The incidence and forms of cheese contamination in the Arab area exhibit significant variations, which may be attributed to several variables like manufacturing practices, governmental control, and consumer awareness. However, some kinds of special milk, such as camel milk, were recorded to be contaminated by toxigenic fungi and their mycotoxins [48]. Filamentous fungi are a common cheese production issue [49]. At the same time, the growth of these fungi on cheese could lead to mycotoxin contamination in manufactured cheese [50,51]. Efforts to enhance food safety standards, advocate for pasteurization, improve storage and distribution infrastructure, and educate producers and consumers may effectively limit the hazards associated with cheese contamination. A range of commitment levels exist to sanitary practices and production standards in cheese manufacture across nations and worldwide areas [52]. In some instances, conventional techniques used to manufacture cheese may fail to adhere to contemporary food safety regulations. Using unpasteurized milk in cheese manufacturing can potentially elevate the likelihood of contamination by pathogenic bacteria, including *E. coli* and *Salmonella*. The pasteurization method, which involves subjecting milk to heat treatment to eliminate harmful microorganisms, is mainly used in industrialized nations to safeguard the integrity and safety of dairy commodities. Traditional cheese production may still include raw milk, which can potentially increase the danger of infection. Ensuring appropriate storage and distribution practices is paramount in mitigating the risk of contamination throughout the supply chain. Cheese contamination may occur when it is subjected to temperature variations or handled unsanitarily [53,54]. Again, the manufacturing steps may give a chance to the occurrence of contamination. Toxigenic fungi and their metabolites still pose a significant hazard for cheese contamination [55]. However, precautions such as safe coating application and natural preservation additives can reduce toxigenic contamination by fungi producing toxins [56,57,58,59].

Cheese contamination with toxigenic fungi was recently reported as a global issue in sour, creamy, semi-hard, soft, and hard cheese [60,61,62]. In the present investigation, toxigenic contamination was recorded as the main issue. Globally, the toxigenic fungal contamination of cheese is linked to the long ripening period and non-hygienic handling conditions. The application of intelligent coating using natural extracts was reported as a successful method to overcome this issue [10,11]. Several extracts were reported to have anti-mycotoxigenic properties [14,63]. These extracts are varied in their sources, from plants or microorganisms.

In contrast, their capacity to reduce toxigenic fungal contamination was also reported. The present investigation was targeted to utilize cell-free or bacterial pellets as active material against toxigenic fungal contamination and their mycotoxins. In the same manner, previous studies investigated some bacteriocin as free or loaded in coating material to reduce the mycotoxigenic risk of contamination in cheese [14,19,64]. Our investigation was based on the activity of bioactive extracts from probiotic bacteria to be applied in the coated cheese material, providing safety characteristics. In the same way, previous studies applied natural extracts of Rosselle [10] or propolis [11] for cheese safety enhancement during storage. Findings of natural extract application in the safe cheese coating were so close to each other; however, in the present investigation, using CFSs and/or bacterial pellets added ameliorative changes regarding the sensory and ripening properties of the cheese.

### 4.1. Characteristic Amelioration of CFS-Coated Cheese

The preferable changes, which were recorded during the cheese ripening, reflected by several parameters amelioration with more acceptability. The relationship between the cheese’s hardness and its moisture content is inverse. Thus, the low hardening values are shown by bacterial coating due to water loss reduction [65]. Also, the proteolytic activity was lowered during ripening, which affected the cheese softening [66]. Furthermore, cheese’s texture relies upon its composition and biochemical changes throughout ripening [2]. The excessive moisture loss in uncoated Ras cheese significantly delays the appearance of their sensory characteristics compared to coated treatments [67]. According to the panelists, the body and texture of uncoated cheese lowered by progressing the ripening period while increasing in different covered cheese treatments. The lowering of moisture of the uncoated cheese acts as a plasticizer in the protein matrix, making it less elastic and not susceptible to fracture [68].

The increase in protein and fat contents in all treatments during storage was mainly linked to moisture loss [61]. These changes may connect to the coating process using chitosan and bacterial CFS contents. The coated cheese proteolysis is slower than uncoated cheese [61]; soluble nitrogen compounds produced through cheese ripening reflect the proteolysis rates. During development, proteolysis hydrolyzes para-casein to polypeptides, breaking down until they reach free amino acids [69]. The previous investigation referred to the ability of lactic acid bacteria to produce substantial proteolytic and lipolytic activities contributing to cheese flavor [70]. The bacteria in the cheese coat contributed to cheese proteolysis, which is evident from the SN value. Previously, the highest protease activity was observed with *L. rhamnosus* PTCC 1637 and *L. fermentum* PTCC 1638 application in cheese [71]. The result regarding these strains’ application reported a protease activity of the CFS lower than of the bacterial cells.

The increase in tryptophan and tyrosine contents throughout the ripening period was expected due to the action of proteolytic enzymes on cheese protein proteolysis. These enzymes could be from rennet or the release of intracellular peptidases from dead and lysed starter cells [72]. The high content of tyrosine and tryptophan in coated cheese with bacteria is due to the contribution of these bacteria to protein breakdown. It plays a significant role throughout ripening, forming flavor [73]. Volatile fatty acids are micro-components that impact cheese flavor and body texture. Total volatile fatty acids linked to the cheese flavor were increased throughout the ripening period for uncoated (control) and coated Ras cheese treatments. The lowest value was recorded related to the coated cheese without bacteria or cell-free supernatant [74].

### 4.2. Ras Cheese Contamination Overcome by Coating

Ras, a traditional cheese consumed in the Middle East, commonly suffers from toxigenic contamination. Application of CFSs or bacterial pellets to the coating material of Ras cheese production is a novel application that may reduce the requirement of preservatives application during ripening storage. Chitosan has more antimicrobial activity against yeasts and molds than Gram-positive and Gram-negative bacteria [75]. Using CFSs or bacterial pellets in a coating process as loaded on chitosan film plays a crucial function in cheese ripening enhancement with preservation properties. These outcomes agree with those obtained by Yangılar [76], who demonstrated that the coating process with chitosan and CWP might have delayed moisture losses compared to the control. Chitosan-based films exhibited excellent barrier properties against water vapor and oxygen [77]. The coating process of cheese, whether for hard, semi-hard, or soft cheese, has been performed to protect it from contamination exposure in direct or cross-contamination during ripening, storage, and handling stages. Earlier investigations have recorded different forms of coating application for the processed cheese, whether in liquid form of coating materials or an external layer of waxy materials applied after the cheese hardening. It is also possible to utilize paper covers supported by preservatives, whether these preservatives are natural or synthetic. For instance, natural extracts were used to coat hibiscus extract with zinc nanoform [10]. Again, nanoemulsion of the propolis extract, loaded on the CMC, was also applied to preserve Kashkaval cheese during maturation and storage [11]. Coated cheese’s lactic acid bacterial count was significantly higher than uncoated cheese’s. He suggested that the presence of coating encourages the growth or survival of those bacteria by lowering permeability to oxygen and increasing the a_w_ [67]. Molds require aerobic conditions; however, yeasts can grow on cheese even under vacuum conditions [78]. The chitosan or chitosan/whey protein-coated Göbek Kashar cheese lowered the action on microbial growth [76].

The formation of coating materials of chitosan emulsion loaded by the CFS and bacterial pellets reflect better characteristics regarding the zeta potential and particle size. The parameter of Zeta potential plays a significant role in the overall stability of the emulsion. In contrast, it will determine whether the particles within an emulsion will tend to aggregate [79]. According to Sharifi et al. [80], nanoemulsion stability could be achieved even with zeta potential less than (+20 mV) or (−20 mV) by changing surface tension, which occurred in the present emulsion due to polysorbate.

### 4.3. Bacterial Functionality and Cheese Safety

The results were shown by an efficiency against *Aspergillus* contamination of coated cheese through the ripening process; also, a reduction in aflatoxin contamination was recorded. Regarding the previous investigations, *A. flavus* showed the lowest mycelia weight compared to other *Aspergillus* species. The mass of the toxigenic mycelia was inhibited by the supernatant of the LAB strains [24,81], demonstrating that *L. rhamnosus* inhibited the abundance of many toxigenic fungi, including species in the genera *Aspergillus* and *Penicillium*. The *L. plantarum* antifungal activity is due to organic acid production, such as lactic, acetic, and phenyl lactic acids [82]. Also, *L. plantarum* MiLAB 393 was reported to produce three antifungal compounds that revealed antifungal effects against various fungi [83]. The fungal strain of *A. niger* was the most tolerant to treatments with LAB cells and CFSs [84].

The amelioration of Ras cheese preservation during ripening storage can mainly be illustrated by binding the bacterial pellets and CFSs loaded on the cheese coating materials. These bioactive molecules are linked to the fungal ability changes concerning the vegetative growth or toxin production ability [15]. The results agree with those reported by Azeem et al. [85], who indicated that the reduced growth and production of aflatoxins by *Aspergillus flavus* subsp. *parasiticus*, and these inhibitory effects may result from lactic acid production or the physical interaction of *lactobacilli* with mold. Also, Chang and Kim [86] demonstrated that extracellular metabolites of *Lactobacillus casei* KC 324 lowered mold growth and aflatoxin production of *Aspergillus flavus* ATCC 15517.

### 4.4. The LAB Application as Antimycotic and Antimycotoxigenic

The loading process of the emulsion composite can ameliorate the cheese characteristics regarding the proximate analysis, maturation, and preservation properties. Probiotic bacteria can inhibit the toxigenic fungi growth in cheese through various mechanisms, including organic acids and antimicrobial compounds, nutrient competition, and immune system modulation. These mechanisms can help to create an unfavorable environment for toxigenic fungi growth, thereby reducing the risk of mycotoxin contamination in cheese. Several studies have investigated the use of probiotic bacteria to prevent toxigenic fungi contamination in cheese [14,29]. For example, *L. plantarum* added to cheese reduced *Aspergillus* growth and limited aflatoxin production [15]. However, adding *L. acidophilus* reduced *Penicillium roqueforti* growth and related mycotoxin production [12]. Application of the CFS of the LAB as a liquid containing bacterial metabolites contained antimicrobial compounds, potentially inhibiting harmful bacteria and molds [87]. The strain *L. plantarum* IS10 produced various antifungal peptides from MRS broth after incubation at 37 °C for 24 h, which inhibited the growth of *A. flavus*. Again, the antifungal activity of different LAB strains was observed to inhibit the *Aspergillus niger* growth [88]. Also, the proteinaceous compound was responsible for the antifungal activity of the CFS from *L. plantarum* YML007 against four molds [89]. By assessing antifungal compounds in *L. plantarum* CFS, three compounds were identified as responsible for the antifungal activity [90].

Besides, *L. rhamnosus* showed a high ability to detoxify aflatoxins [91]. Its cell-free extract can interact with AFB_1_ with a high degradation percentage [92]. Moreover, *L. rhamnosus* LBGG and LC-705 achieved 80% removal of AFB_1_ from liquid media [93]. The reduced aflatoxin production by LAB is illustrated by their released molecules, which inhibit fungal growth and reduce mycotoxin production [94]. Also, the bioactive compounds secreted by LAB, such as organic acids, Phenyllactic, reuterin, fatty acids, peptides, and cyclic peptides, can interact with aflatoxins, reducing their amount [95].

Based on the previous results, *L. rhamnosus* CFS represents an efficient antifungal properties strain among the investigated strains. This CFS is chosen to provide various benefits over standard preservatives in food preservation [14]. CFSs are non-toxic, natural preservatives with no harmful effects associated with synthetic preservatives. Also, CFSs are derived from bacterial cultures already used in food production, such as lactic acid bacteria, making them a clean category ingredient [15]. The particular bioactive chemicals available in the extract might vary based on the bacterial strain, growing circumstances, and extraction process, which is one possible restriction of CFSs.

## 5. Conclusions

Ras cheese samples collected from local markets showed significant contamination levels, particularly with toxin-producing fungi. These results prompted the authors to search for promising methods to overcome mycotoxin contamination on hard cheese during ripening and storage. Bacterial cell-free extracts were able to reduce the growth of toxigenic fungi using two assays (disc and well diffusion). These results were confirmed by estimating the decrease in dry mycelial weight of fungi grown in liquid media (with or without cell-free extract). The results of evaluating the cell-free extract indicated the effectiveness of Bifidobacteria and *L. halvaticus* strains as antifungal with the ability to inhibit growth. At the same time, the efficiency of *L. rhmnosus* was average.

On the contrary, *L. rhmnosus* showed a significant reduction in the toxigenic fungal ability to produce aflatoxins in a liquid growth media. In evaluating coating material consisting of chitosan loaded with either cell-free extract or bacterial cell pellet, the results showed a clear distinction and quality of the nanoemulsion solution prepared for coating cheese. The results also reflected the stability of the resulting emulsion, which supports its functional application in cheese preservation by coating. The evaluating cheese coated with chitosan-containing cell extract showed a significant amelioration in rheological properties and ripeness characteristics, in addition to better acceptance for the sensory evaluation of the cheese. Again, applying bacterial LCP or CFS to cheese coating enhances its proximate analysis, ripening parameters, and microbiological properties. Overall, using CFS and LCP in food preservation is a promising food application that provides a natural alternative to synthetic preservatives, which may improve food products’ sensory and nutritional properties. When evaluating the apparent contamination of the surface of the coated cheese, the results showed an excellent ability to reduce contamination with Aspergillus fungi during storage. The use of probiotics in cheese against toxic fungi is a promising approach. Therefore, further studies are needed to fully understand the mechanisms of action of cell granules when applied alone or with bacterial cell-free extract.

## Figures and Tables

**Table 1:** Characteristics estimated for the control and loaded films utilized in cheese coating.

Sample	PSS (nm)	ZPV (mV)	PDI	ES (%)
F1	102.84 ± 4.18	8.21 ± 1.56	0.37 ± 0.11	91.51 ± 1.02
F2	127.37 ± 5.41	11.34 ± 1.02	0.42 ± 0.05	88.26 ± 0.87
F3	197.66 ± 7.21	9.66 ± 2.05	0.49 ± 0.04	85.61 ± 1.37
F4	218.37 ± 5.73	12.48 ± 1.18	0.44 ± 0.05	89.34 ± 1.41

Results expressed as means ±SD (standard division; n = 5; *p* = 0.05). PSS: particle size in nanometer; ZPV: zeta potential value expressed in millivolt; PDI: poly dispersing index; ES: emulsion stability measured in percentage for the non-separated emulsion. F1: coated material without loading (control film); F2: coated material loaded by bacterial-cell pellet; F3: coated material loaded by cell-free extract; F4: coated material loaded by the mix of LCP and CFS.

**Table 2:** Chemical composition (% *w*/*w*) of Ras cheese.

Sample	RP (Days)	Moisture	Fat	F/DM	Protein	Salt	Acidity	pH
C	zero	46.53 ± 1.70	24.00 ± 0.79	44.88 ± 1.39	17.47 ± 0.56	1.87 ± 0.16	1.98 ± 0.26	5.34 ± 0.06
	30	41.32 ± 2.00	29.00 ± 0.68	49.42 ± 1.71	21.85 ± 0.75	2.22 ± 0.15	1.98 ± 0.18	5.26 ± 0.08
	60	37.36 ± 1.94	32.00 ± 0.64	51.09 ± 1.39	23.93 ± 0.94	3.25 ± 0.14	2.01 ± 0.16	5.28 ± 0.06
	90	33.92 ± 1.45	33.45 ± 0.85	50.62 ± 1.00	25.15 ± 1.43	3.75 ± 0.15	2.02 ± 0.26	5.19 ± 0.07
	120	30.32 ± 2.34	34.00 ± 0.73	48.79 ± 0.39	26.87 ± 0.79	4.91 ± 0.17	2.20 ± 0.31	5.18 ± 0.05
T1	zero	45.90 ± 3.48	24.50 ± 0.77	45.29 ± 1.30	17.67 ± 0.68	1.88 ± 0.23	1.86 ± 0.25	5.47 ± 0.11
	30	41.70 ± 2.25	28.80 ± 0.52	49.40 ± 1.94	21.44 ± 0.50	2.30 ± 0.21	1.88 ± 0.32	5.38 ± 0.08
	60	37.52 ± 2.29	32.30 ± 0.73	51.70 ± 1.89	23.95 ± 0.75	3.23 ± 0.10	1.89 ± 0.17	5.39 ± 0.05
	90	36.80 ± 2.36	33.10 ± 0.65	52.37 ± 1.44	24.33 ± 0.90	3.55 ± 0.21	2.00 ± 0.28	5.14 ± 0.06
	120	34.20 ± 1.66	35.00 ± 0.84	53.19 ± 2.54	26.04 ± 0.15	4.20 ± 0.19	2.10 ± 0.29	5.16 ± 0.06
T2	zero	46.57 ± 3.17	24.70 ± 0.78	51.84 ± 1.24	17.47 ± 0.80	1.84 ± 0.06	1.86 ± 0.15	5.44 ± 0.04
	30	41.00 ± 1.54	29.00 ± 1.02	49.15 ± 2.07	19.43 ± 0.60	2.27 ± 0.12	1.87 ± 0.21	5.38 ± 0.07
	60	38.99 ± 1.70	30.10 ± 0.55	49.34 ± 2.77	20.01 ± 0.75	3.25 ± 0.15	1.99 ± 0.25	5.16 ± 0.07
	90	35.97 ± 1.97	32.20 ± 0.55	50.29 ± 1.96	23.15 ± 0.61	4.20 ± 0.11	2.00 ± 0.26	5.16 ± 0.02
	120	32.30 ± 2.31	35.50 ± 1.07	52.43 ± 0.83	25.40 ± 0.84	4.65 ± 0.18	2.3 ± 0.17	5.13 ± 0.08
T3	zero	46.75 ± 1.79	24.40 ± 0.68	45.82 ± 1.42	17.42 ± 1.18	1.85 ± 0.23	1.87 ± 0.23	5.34 ± 0.11
	30	41.32 ± 1.62	28.60 ± 0.86	48.74 ± 1.60	21.80 ± 1.33	2.21 ± 0.28	2.00 ± 0.30	5.28 ± 0.14
	60	39.50 ± 2.77	30.60 ± 0.81	50.58 ± 1.68	22.99 ± 0.74	3.81 ± 0.15	2.01 ± 0.24	5.28 ± 0.07
	90	36.20 ± 2.29	31.90 ± 0.67	50.00 ± 0.79	24.53 ± 0.90	3.99 ± 0.18	2.01 ± 0.25	5.17 ± 0.02
	120	34.51 ± 1.85	36.40 ± 0.83	55.58 ± 1.43	25.49 ± 1.37	4.50 ± 0.19	2.12 ± 0.26	5.14 ± 0.04
T4	zero	46.69 ± 2.28	24.60 ± 0.85	46.15 ± 1.18	17.46 ± 0.83	1.84 ± 0.22	1.88 ± 0.27	5.51 ± 0.04
	30	41.30 ± 3.47	29.90 ± 1.04	50.94 ± 1.96	21.80 ± 0.62	2.21 ± 0.17	1.99 ± 0.22	5.36 ± 0.08
	60	39.70 ± 2.16	30.50 ± 0.98	50.58 ± 2.03	22.95 ± 0.64	3.24 ± 0.12	2.00 ± 0.21	5.34 ± 0.02
	90	35.90 ± 2.83	31.70 ± 1.02	49.45 ± 2.08	24.40 ± 0.67	3.97 ± 0.12	2.01 ± 0.27	5.31 ± 0.02
	120	35.65 ± 2.84	33.20 ± 0.69	51.59 ± 0.81	25.44 ± 0.64	4.41 ± 0.11	2.17 ± 0.20	5.15 ± 0.01

Results were expressed as means ±SD (standard division; n = 5; *p* = 0.05). RP: cheese period stored conditionally for ripening; CFS: cell-free extract of bacteria; LCP: bacterial cell pellet. C: control cheese, T1: cheese coated with plain film (a raw film without loading); T2: cheese coated with LCP; T3: cheese coated with CFS; T4: cheese coated with CFS-LCP.

**Table 3:** Indices of ripening in Ras cheese during the ripening period.

Sample	RP (Days)	SN (%)	TVFA	Tryptophan [mg/100 g]	Tyrosine [mg/100 g]
C	zero	0.23 ± 0.06	8.20 ± 0.68	3.17 ± 1.78	7.50 ± 2.19
	30	0.35 ± 0.08	24.70 ± 0.40	14.75 ± 1.25	34.10 ± 1.98
	60	0.52 ± 0.06	29.00 ± 0.71	22.10 ± 1.15	52.30 ± 1.90
	90	0.63 ± 0.07	38.00 ± 1.19	49.56 ± 1.46	92.23 ± 1.45
	120	0.74 ± 0.05	63.50 ± 0.70	68.10 ± 0.71	123.60 ± 2.50
T1	zero	0.22 ± 0.11	8.00 ± 0.82	3.40 ± 1.07	7.10 ± 3.83
	30	0.33 ± 0.08	19.10 ± 0.64	14.98 ± 1.63	33.89 ± 3.13
	60	0.41 ± 0.05	31.00 ± 0.94	22.45 ± 2.21	53.45 ± 3.22
	90	0.47 ± 0.06	38.80 ± 1.05	35.28 ± 1.15	80.66 ± 2.84
	120	0.57 ± 0.06	54.60 ± 0.25	57.55 ± 2.89	109.97 ± 3.20
T2	zero	0.26 ± 0.04	7.80 ± 0.98	3.35 ± 1.07	7.37 ± 3.54
	30	0.37 ± 0.07	26.90 ± 0.66	14.85 ± 1.75	34.47 ± 1.91
	60	0.44 ± 0.07	35.50 ± 0.87	23.84 ± 2.46	53.94 ± 3.47
	90	0.60 ± 0.02	42.60 ± 0.31	46.12 ± 1.68	88.81 ± 2.09
	120	0.72 ± 0.08	67.80 ± 0.72	68.09 ± 1.56	120.36 ± 2.28
T3	zero	0.26 ± 0.11	8.10 ± 1.32	3.26 ± 1.73	7.80 ± 2.32
	30	0.34 ± 0.14	24.10 ± 1.35	15.00 ± 2.00	35.04 ± 1.70
	60	0.44 ± 0.07	30.2 ± 0.84	22.99 ± 2.07	54.88 ± 3.44
	90	0.49 ± 0.02	40.42 ± 0.81	40.75 ± 1.13	84.96 ± 2.29
	120	0.55 ± 0.04	62.20 ± 1.22	60.72 ± 1.84	114.52 ± 1.84
T4	zero	0.25 ± 0.04	7.90 ± 0.75	3.06 ± 1.89	7.95 ± 3.36
	30	0.34 ± 0.08	32.90 ± 0.76	14.56 ± 2.73	36.52 ± 2.70
	60	0.46 ± 0.02	39.60 ± 0.64	24.56 ± 1.70	55.12 ± 2.23
	90	0.52 ± 0.02	46.00 ± 0.81	44.66 ± 1.75	86.12 ± 3.80
	120	0.68 ± 0.01	69.60 ± 0.76	65.50 ± 1.19	117.20 ± 2.87

Results were expressed as means ± SD (standard division; n = 5; *p* = 0.05). RP: the period that cheese is stored conditionally for the ripening; SN: soluble nitrogen determined as a percentage ratio. TVFA: total volatile fatty acids [determined as mL 0.1 N NaOH/100 g cheese]. C: control cheese, T1: cheese coated with plain film (a raw film without loading); T2: cheese coated with LCP; T3: cheese coated with CFS; T4: cheese coated with CFS-LCP.

**Table 4:** Rheological analysis of treated Ras cheese samples during ripening storage.

Treatment	RP (Days)	Hardness (N)	Adhesiveness (mj)	Cohesiveness (Ratio)	Springiness (mm)	Gumminess (N)	Chewiness (mj)
	Fresh	25 ± 0.26	0.494 ± 0.20	0.22 ± 0.25	3.23 ± 0.52	13.29 ± 0.20	74.34 ± 3.22
Control	30	41.8 ± 0.18	0.280 ± 0.22	0.57 ± 0.20	4.72 ± 0.24	15.75 ± 0.48	98.12 ± 4.08
	60	60.4 ± 0.21	0.256 ± 0.22	0.60 ± 0.17	5.23 ± 0.52	28.84 ± 0.42	291.02 ± 3.90
	90	73.5 ± 0.26	0.170 ± 0.19	0.63 ± 0.26	6.76 ± 0.45	44.2 ± 0.46	344.77 ± 5.41
	120	91.8 ± 0.31	0.148 ± 0.20	0.69 ± 0.32	7.40 ± 0.49	52.3 ± 0.28	420.92 ± 1.61
T1	Fresh	21.4 ± 0.25	0.500 ± 0.32	0.22 ± 0.29	3.23 ± 0.15	12.00 ± 0.28	34.27 ± 3.26
	30	34.3 ± 0.32	0.466 ± 0.26	0.36 ± 0.28	4.45 ± 0.23	13.78 ± 0.21	90.31 ± 4.67
	60	59.2 ± 0.27	0.240 ± 0.49	0.46 ± 0.47	5.66 ±0.03	25.32 ± 0.21	172.64 ± 3.30
	90	70.12 ± 0.28	0.169 ± 0.15	0.52 ± 0.15	6.59 ± 0.17	41.00 ± 0.28	270.24 ± 4.68
	120	87.2 ± 0.29	0.126 ± 0.40	0.60 ± 0.14	6.67 ± 0.23	49.90 ± 0.15	298.62 ± 4.76
T2	Fresh	22.6 ± 0.15	0.522 ± 0.08	0.23 ± 0.28	3.44 ± 0.40	12.00 ± 0.53	33.12 ± 2.88
	30	33.2 ± 0.21	0.298 ± 0.86	0.33 ± 0.24	4.32 ± 0.52	11.81 ± 0.12	85.76 ± 5.35
	60	59.7 ± 0.25	0.256 ± 0.20	0.42 ± 0.25	5.36 ± 0.06	21.59 ± 0.20	150.37 ± 4.69
	90	71.56 ± 0.26	0.171 ± 0.24	0.46 ± 0.24	6.44 ± 0.52	38.00 ± 0.44	233.28 ± 3.81
	120	83.45 ± 0.17	0.116 ± 0.12	0.57 ± 0.19	6.28 ± 0.40	43.65 ± 0.23	260.96 ± 4.29
T3	Fresh	26.6 ± 0.23	0.533 ± 0.16	0.25 ± 0.35	3.34 ± 0.23	13.92 ± 0.26	36.69 ± 3.30
	30	39.8 ± 0.30	0.398 ± 0.35	0.48 ± 0.17	4.44 ± 0.17	14.10 ± 0.14	89.81 ± 5.40
	60	62.44 ± 0.24	0.238 ± 0.56	0.49 ± 0.32	5.65 ± 0.15	24.22 ± 0.17	164.71 ± 4.57
	90	73.58 ± 0.25	0.190 ± 0.34	0.57 ± 0.36	6.60 ± 0.23	40.11 ± 0.21	245.78 ± 2.76
	120	87.80 ± 0.26	0.125 ± 1.11	0.65 ± 0.20	6.69 ± 0.32	45.51 ± 0.32	357.18 ± 6.15
T4	Fresh	23.2 ± 0.27	0.512 ± 0.13	0.25 ± 0.36	3.22 ± 0.48	13.00 ± 0.20	35.62 ± 1.64
	30	32.02 ± 0.22	0.284 ± 0.34	0.34 ± 0.42	4.30 ± 0.45	11.59 ± 0.27	85.11 ± 2.52
	60	68.20 ± 0.21	0.216 ± 0.56	0.45 ± 0.25	5.29 ± 0.52	20.92 ± 0.12	148.07 ± 3.83
	90	72.15 ± 0.27	0.168 ± 0.50	0.54 ± 0.35	6.49 ± 0.49	38.93 ± 0.23	230.26 ± 3.35
	120	84.70 ± 0.20	0.105 ± 0.22	0.62 ± 0.27	6.35 ± 0.14	42.82 ± 0.20	255.64 ± 4.29

Results were expressed as means ±SD (standard division; n = 5; *p* = 0.05). RP: the period that cheese is stored conditionally for ripening. C: control cheese, T1: cheese coated with plain film (a raw film without loading); T2: cheese coated with LCP; T3: cheese coated with CFS; T4: cheese coated with CFS-LCP.

**Table 5:** Microbiological changes (log 10 CFU/g) of uncoated and different coated Ras cheese treatments.

Sample	RP (Days)	TBC	Y and M	Lipolytic Bacteria	Proteolytic Bacteria
C	zero	7.217 ± 1.25	-	4.332 ± 1.78	4.191 ± 1.26
	30	7.832 ± 1.13	2.144 ± 0.40	4.522 ± 1.25	4.361 ± 1.18
	60	8.251 ± 0.67	3.786 ± 0.71	4.230 ± 1.15	4.491 ± 1.16
	90	7.564 ± 1.30	4.701 ± 1.19	4.079 ± 1.46	4.681 ± 1.26
	120	6.380 ± 0.79	5.698 ± 0.70	3.698 ± 0.71	3.602 ± 1.31
T1	zero	7.200 ± 0.77	-	4.321 ± 1.07	4.195 ± 1.25
	30	7.869 ± 0.70	-	4.518 ± 1.63	4.493 ± 1.32
	60	8.361 ± 1.11	3.903 ± 0.94	4.361 ± 2.21	4.579 ± 1.17
	90	7.702 ± 0.70	4.677 ± 1.05	4.114 ± 1.15	4.613 ± 1.28
	120	6.490 ± 1.27	4.402 ± 0.25	3.845 ± 2.89	3.602 ± 1.29
T2	zero	7.209 ± 0.77	-	4.361 ± 1.07	4.255 ± 1.15
	30	7.948 ± 0.95	-	4.556 ± 1.75	4.447 ± 1.21
	60	8.505 ± 0.69	3.301 ± 0.87	4.342 ± 2.46	4.518 ± 1.25
	90	7.839 ± 0.70	3.954 ± 0.31	4.154 ± 1.68	4.690 ± 1.26
	120	6.541 ± 1.46	4.177 ± 0.72	3.898 ± 1.56	3.698 ± 1.17
T3	zero	7.206 ± 1.13	-	4.322 ± 1.73	4.114 ± 1.08
	30	7.890 ± 1.30	-	4.477 ± 2.00	4.447 ± 0.96
	60	8.414 ± 1.26	-	4.342 ± 2.07	4.491 ± 1.24
	90	7.770 ± 1.12	4.278 ± 0.81	4.079 ± 1.13	4.653 ± 1.06
	120	6.514 ± 1.27	4.407 ± 1.22	3.745 ± 1.84	3.954 ± 1.06
T4	zero	7.201 ± 0.82	-	4.325 ± 1.89	4.276 ± 1.27
	30	7.960 ± 0.96	-	4.579 ± 2.73	4.397 ± 1.22
	60	8.520 ± 0.91	-	4.462 ± 1.70	4.577 ± 1.21
	90	7.780 ± 0.98	-	4.146 ± 1.75	4.716 ± 0.83
	120	6.549 ± 1.14	3.477 ± 0.76	3.903 ± 1.19	3.778 ± 1.20

Results were expressed as means ±SD (standard division; n = 5; *p* = 0.05). RP: the period that cheese is stored conditionally for ripening. C: control cheese; T1: cheese coated with plain film (raw film without loading); T2: cheese coated with LCP; T3: cheese coated with CFS; T4: cheese coated with CFS-LCP.

## Data Availability

All data were available in the present manuscript.

## Supplementary Materials

The following supporting information can be downloaded at: https://www.mdpi.com/article/10.3390/foods12193548/s1, Table S1: Microbial counts of collected cheese samples represented in log CFU/g; Figure S1: Uncoated and coated Ras cheese on the end ripening period.
