# Supplementary material for: Neoteric Biofilms Applied to Enhance the Safety Characteristics of Ras Cheese during Ripening
Source: Foods. 2023 Sep 24;12(19):3548. doi: 10.3390/foods12193548 (PMC10572299; doi:10.3390/foods12193548)
Supplement: Supplementary file 1 [file foods-12-03548-s001.zip › foods-2595485-supplementary.pdf]

Table (S1): Microbial counts of collected cheese samples represented in log CFU/g

	Bacterial count **(Log CFU/g)**					
Sample area	*E. coli*	*Staph. aureus*	*S.typhi*	*B. subtilis*	*L. lactis*	*L. bulgaricus*
West Delta	2.16±0.67	0.95±0.008	0.61±0.033	1.52±0.071	2.49±0.26	2.39±0.37
East Delta	1.23±0.56	1.22±0.25	0.5±0.02	1.09±0.02	2.47±0.17	2.28±0.56
Middle Delta	0.82±0.11	1.63±0.33	nd	1.45±0.25	2.16±0.42	2.48±0.45
Great Cairo	1.48±0.42	1.37±0.56	nd	1.20±0.33	2.42±0.31	2.47±0.33
Upper Egypt	0.92±0.09	2.12±0.45	nd	0.48±0.09	2.43±0.41	2.17±0.42
	Fungal count **(Log CFU/g)**					
Sample area	*A.flavus*	*A.paraciticus*	*A. nominos*	*A. niger*	*P.chrysoginum*	*P. notatum*
West Delta	2.10±0.13	2.62±0.27	1.72±0.18	2.06±0.17	1.93±0.31	2.06±0.12
East Delta	2.46±0.28	2.74±0.08	nd	2.09±0.21	1.94±0.16	2.03±0.16
Middle Delta	2.80±0.22	2.77±0.25	1.51±0.11	2.09±0.05	2.04±0.11	2.08±0.33
Great Cairo	2.47±0.14	2.52±0.14	1.79±0.14	2.15±0.24	2.08±0.14	2.03±0.14
Upper Egypt	2.86±0.28	2.76±0.02	nd	2.12±0.15	1.89±0.05	1.84±0.22

- The result were expressed in log CFU/g (mean ± SD; n = 5; *p* = 0.05)
- nd: represent not detected microbes in the examined samples.

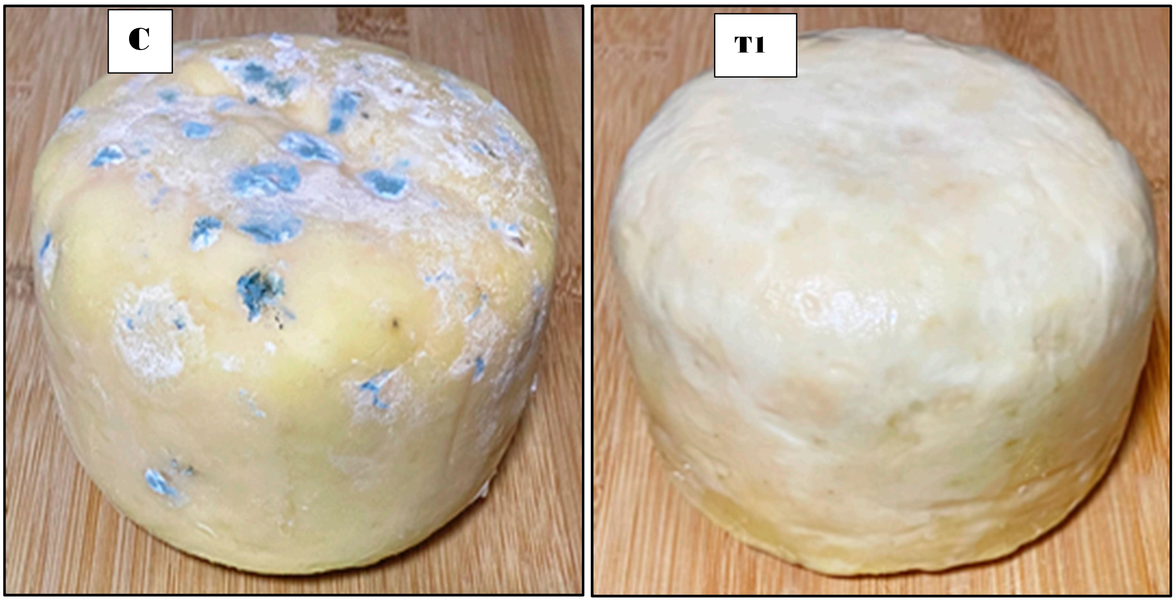

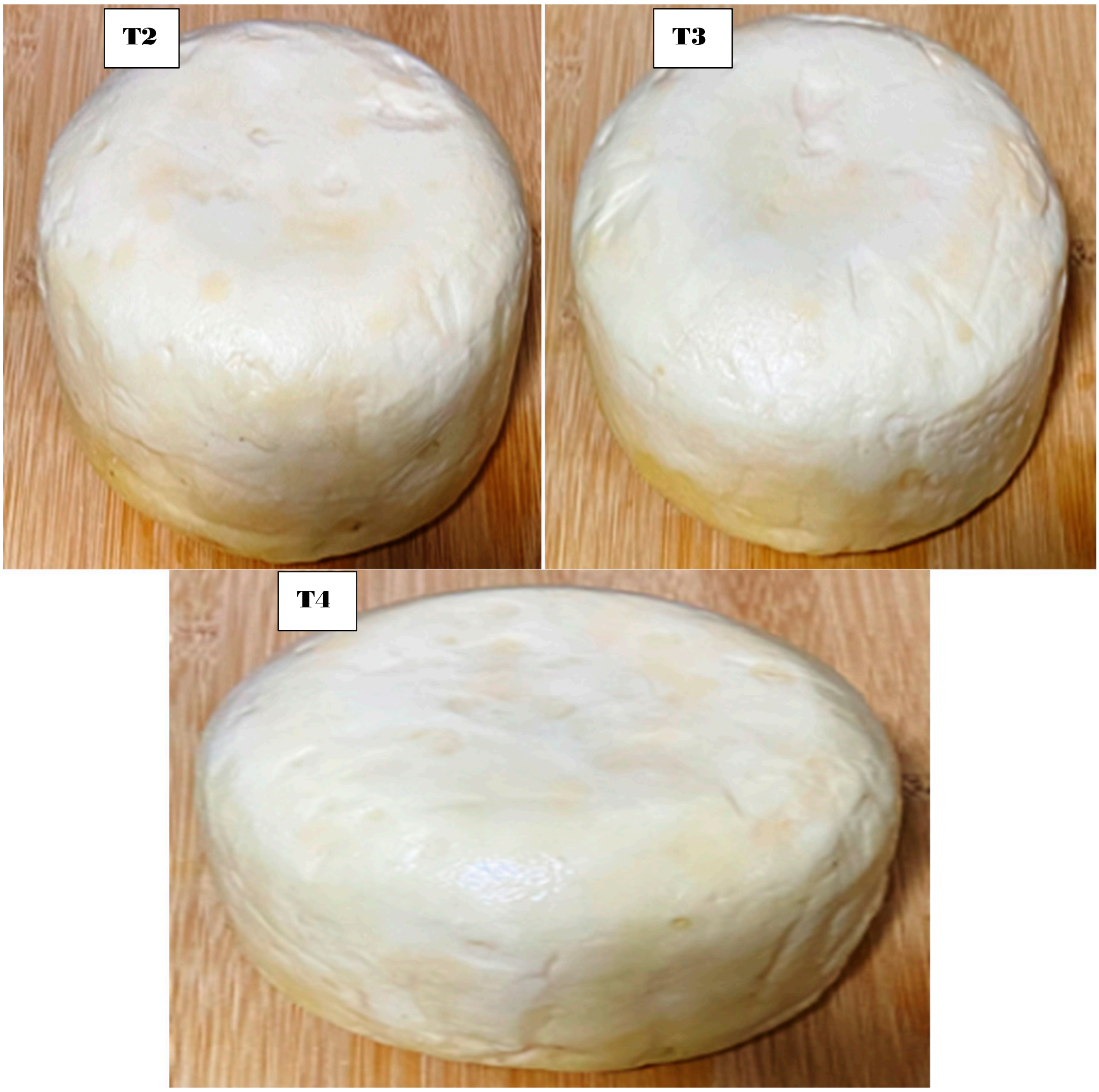

Figure S1: Uncoated and coated Ras cheese on the end ripening period

- C: control cheese, T1: cheese coated by the plain film (a raw film without loading); T2: cheese coated with LCP; T3: cheese coated with CFS; T4: cheese coated with CFS-LCP
